# The insects as food and feed industry needs integrative solutions

**DOI:** 10.1016/j.cris.2026.100127

**Published:** 2026-06-06

**Authors:** Jacinta D. Kong, Matthew J. Muzzatti, Catherine I. Cullingham, Vivian Hoffmann, Susan M. Bertram, Heath A. MacMillan

**Affiliations:** a Department of Biology, Carleton University, Ottawa, K1S 5B6, Canada; b Department of Entomology, College of Agriculture and Life Sciences, Cornell University, Ithaca, NY 14850, USA; c Department of Economics, Carleton University, Ottawa, K1S 5B6, Canada

**Keywords:** Insect agriculture, Entomophagy, Food security

## Abstract

The edible insect industry is inherently multidisciplinary. The increasing interest in edible insect production for food and feed has driven an increasing number of research outputs covering biology, engineering, economics, and more. However, research spanning disciplines lags behind discipline-specific research and there is a need for interdisciplinary research to address emerging challenges and support industrial advancement. Here, we integrate perspectives across biology and economics to highlight opportunities for interdisciplinary research across these fields. Specifically, we identify key gaps requiring collaborative effort and explicitly outline solutions that span these fields, with the aim of scaling up production yield, achieving economic sustainability, and ensuring the sustainable development of edible insect farming. Continuing to build relationships within academia, and between industries and governments, will ensure the continuous development of the edible insect industry to meet global challenges of hunger and food security.

## Highlights

• Discipline-specific research is siloed in edible insect literature.
• Interdisciplinary research fosters industrial advancement to meet key challenges.
• Shared industry goals drive collaborative synergy across disciplines.
• Building partnerships among academia, industry and governments is crucial.

## Introduction: A problem of scale

Insects provide an alternative source of protein for human consumption and for conventional agriculture. Eating insects, or entomophagy, is practiced globally with an estimated 1600 to over 2000 species of insects and related terrestrial arthropods traditionally eaten (Liceaga et al., 2022; Van Itterbeeck and Pelozuelo, 2022). In addition to food for human consumption, insects are added as a feed supplement for raising fish (Hua, 2021), chicken (Leiber et al., 2017), and swine (Hong and Kim, 2022). Alternative products from insects also include fertiliser derived from frass (Beesigamukama et al., 2022), non-protein bioactive compounds (D'Antonio et al., 2021), or fermentation products (Castro-López et al., 2020). Insects are further used to process by-products or organic waste from other sources, facilitating a circular economy (van Huis and Oonincx, 2017; Moruzzo et al., 2021). These end products or services are variable in production, processing, and distribution (Hawkey et al., 2021). This results in an edible insect industry supply chain that is complex and interdisciplinary, drawing expertise from disparate fields including evolutionary biology, biotechnology, and economics to name a few.

History, needs, and local culture have driven region-specific development of the edible insect industry across Africa (Tanga et al., 2021), Australia (Ponce Reyes and Lessard, 2023), North America (Larouche et al., 2023), Europe (Żuk-Gołaszewska et al., 2022), and Latin America (Bermúdez-Serrano, 2020). These differences have consequences for the choice and diversity of insects produced or harvested (Raheem et al., 2019), production methods and market supply, and the degree of insect protein integration into agricultural feed (Kim et al., 2019). For example, the degree of industrialisation varies from hand harvesting of wild-caught insects using traditional knowledge, to small scale production of many species, to high-tech mass-production specialising in one species (Wade and Hoelle, 2020; Robinson et al., 2024). Regardless of region, a common problem in the edible insect industry is scale. Small scale or traditional production cannot meet global market demands, and mass-production of insects currently cannot compete with dominant commercial sources of protein such as fishmeal and soymeal (Biteau et al., 2024).

Three overarching goals to develop the industry have emerged from this general problem of scale: 1) to increase yield (scale-up production) at minimal cost (González and Neff, 2026), 2) to ensure long-term economic sustainability and competitiveness (Biteau et al., 2024), and 3) to ensure industry growth aligns within environmental sustainability and sustainable development principles (e.g. UN SDGs (Oonincx and de Boer, 2012; Moruzzo et al., 2021; Tanga et al., 2021)). Research has addressed parts of these goals within siloed disciplined areas. However, the edible insect industry is multidisciplinary and the solutions to achieve these goals are also multidisciplinary. Thus, there is an opportunity for interdisciplinary research that synthesises knowledge across fields to address common industry goals but such research lags behind siloed discipline-specific research.

**Fig. 1:**
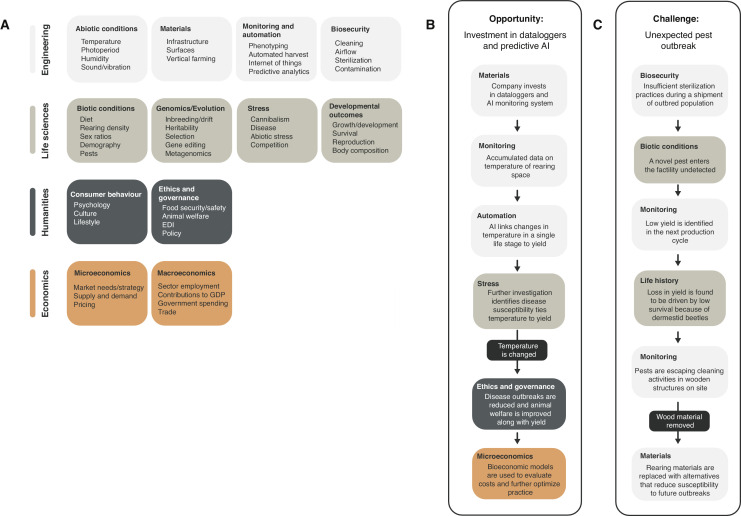
A) Mass-rearing of edible insects is highly multidisciplinary and interdisciplinary but research areas are siloed within their respective disciplines (colours). Examples of specific areas of interest within each discipline are provided. EDI: Equity, Diversity & Inclusion. GDP: gross domestic product. Hypothetical examples of how different disciplines (colours) are invoked to B) address an opportunity for automation and artificial intelligence (AI) in insect farming, and C) detect and respond to an unexpected pest outbreak.

In this review, we highlight opportunities and challenges that complement and synergise research into edible insects towards the three overarching goals of the industry.Specifically, we discuss how biological insights (e.g. nutritional ecology, stress response physiology), technological innovations (e.g. precision agriculture), and economic modelling (e.g. cost-benefit analyses) intersect with a focus on highly industrialised mass-rearing (Fig. 1A). This form of insect production, common in North America and Europe, favours insects with small sizes, short generation times, and tolerance of high-density populations that can be continuously produced (Larouche et al., 2023; Robinson et al., 2024). Thus, this sector is centred around a few candidate species that were already mass-reared for other purposes (e.g. pet food), and that can strongly benefit from species-specific research and development (Davidowitz, 2021). Such research may not directly translate to all forms of insect production (e.g. wild-caught harvesting or apiaries) but there are fundamental principles and concepts about insects and mass-rearing that unite all insect production and generate overarching goals. This is an opportune time for future scoping of the edible insect industry to guide research and development towards a sustainable future.

### Engineering the abiotic environment to influence insect performance

The abiotic environment includes several components (temperature, water, light, sound, etc.) that strongly influence insect performance in ways that can be beneficial or detrimental towards mass-rearing. This interaction feeds into other biological or industrial aspects of mass-rearing. Individual insect performance sets a foundation for the performance of the population, which is a critical component of yield (Kong et al., 2025; Muzzatti et al., 2025). Most insects are ectotherms, meaning environmental temperature will strongly and directly impact an individual’s performance, typically in non-linear ways. Temperature can have positive impacts on insect performance and harvest yield within a given range but negative impacts at the extremes (Kong et al., 2025). The small body sizes of insects that make them ideal for mass-rearing also makes them suspectable to desiccation and dehydration. Levels and timing of light exposure can also strongly impact insect performance as light drives circadian patterns of life history. These characteristics mean small changes to the abiotic environment can have disproportionate consequences on yield for insect mass-rearing, giving strong rationale for engineering solutions for controlling and manipulating abiotic environments.

Understanding the relationships between insects and their abiotic environment in the laboratory has a big role to play in identifying optimal environmental conditions for rearing. Optimal rearing environments have the potential to improve yield by minimising detrimental effects on insect life history. That said, population-level performance also critically depends on the social environment and broader context in which it is studied. A growing number of studies target either individual performance or group performance (e.g. at production scale), but a mechanistic understanding that connects between these levels is needed to inform innovative solutions to unexpected outcomes in the longer term (Muzzatti et al., 2025). For example, optimal farming temperatures studied at a small scale may suggest an optimal temperature below those that are optimal at the individual scale because insect and microbial biomass generates additional heat (Li et al., 2023). These unintended consequences of scale can be revealed through co-operative experiments at multiple scales with industrial partners.

Control of the abiotic environment for insect rearing varies globally. Farming in semi-open and open facilities where insects are exposed to natural environmental temperatures is common in areas where there is comparatively less daily or seasonal environmental variation. In contrast, insects in closed facilities are isolated from the outside environment under highly-controlled and constant abiotic environments that can be highly engineered (Eilenberg and Jensen, 2017). The open approach is sensible and low-cost when the mass-reared species is of tropical origin, but we poorly understand how more natural conditions like diurnal temperature cycles may drive benefits, or how unexpected events (e.g. failure of temperature control systems on a hot day causing a heat spike) can cause immediate or latent harms to production, even under controlled settings (Lee et al., 2024).

Insects are adept at sensing and responding to changes in their thermal environment in space and time. Behavioural thermoregulation is part of how insects maintain homeostasis by selecting microclimates that can benefit physiological functions (Gardner et al., 2024). Few mass-rearing systems allow insects to choose preferential microclimates, but specifically integrating spatial variation in temperature into rearing facilities through heated or sheltered areas could leverage natural insect behaviours for thermoregulation, potentially optimising growth and reducing mortality without significant increases in energy costs. Of course, behaviours could also limit yield if chosen microenvironments do not improve all relevant traits of interest (Leith et al., 2024). We know little about the role of behaviours in responses to abiotic conditions beyond model organisms or how these interactions occur in mass-rearing systems. This information can reveal factors that can be leveraged towards improving yields or considering animal welfare (Lambert et al., 2021).

Liquid water is freely provided in many farming systems for insects to drink. However, excess water availability can be problematic regardless of the rearing medium. An abundance of water can lead to product loss directly through drowning. Improper air flow or high rearing density, in concert with high temperatures can indirectly lead to excess humidity that may drive other negative environmental effects, like growth of harmful microbes or viral disease (Szelei et al., 2011; Herren et al., 2023). Beyond practical implications, the water balance of insects drives important physiological processes of osmoregulation and desiccation that can intersect with how insects respond to temperature stress but the responses of insect to water stressors are understudied compared with temperature stressors.

Insects are also sensitive to sound and substrate-borne vibrations. Sound and vibration are key factors used in insect communication and can carry important informational signals (Virant-Doberlet et al., 2023). In a mass-rearing environment, false cues of danger that mimic predators in the form of vibrations from nearby infrastructure (e.g. roads), equipment, staff, or pests may drive insects to exhibit startle responses leading to inactivity, escape behaviour, or increased stress. All of these responses can divert time and resources away from growth (Eriksson et al., 2012). Industrial-scale mass-rearing environments, particularly those operating with robotics and product processing machinery, can be consistently noisy places, and anthropogenic noise is known to impact how insects detect and respond to auditory signals (Classen-Rodríguez et al., 2021). Careful facility design that shields insects from the noise generated by industrial facilities could mitigate the risk of detrimental effects on insects, and specific testing of these effects can occur through co-operations between entomologists and engineers. Mitigation strategies for loud machinery would also benefit the health and safety of workers in these spaces.

Regardless of what stressors an insect experiences, the timing of stressor application can strongly influence its impacts. Stressors experienced early in life can lead to carry-over effects or physiological responses that impact production in ways that are difficult to predict (Pechenik, 2006). For example, rearing environment can influence heat and cold resistance in fruit flies, and the impacts of a thermal stress in one generation can affect the next generation differently, depending on the species (Schiffer et al., 2013). The timing of stressors is also relevant for other potential stressors such as suboptimal diets. Crickets fed low quality food early in life respond by delaying maturity and reach the same final body size (Kelly et al., 2014). Such an event could impact harvest schedules or yield, depending on production constraints.

### Diet is an economic and biological problem

Designing an artificial diet for insect mass-production is challenging; it should align with the insect’s nutritional needs, be appealing to the insect, and remain economical, using cost-effective ingredients that support the economics of large-scale production (Morales-Ramos et al., 2020). A goal unique to edible insects is to design diets that improve the nutritional quality of insects for other animals (Kolobe et al., 2023). Decades of nutritional ecology research have identified the relative availability of protein to carbohydrate as the most important factor in how diet shapes insect life history, and this framework has been applied to edible insects (Cheon et al., 2022; Muzzatti et al., 2024). However, a key difference between nutritional ecology and dietary studies for edible insects is the choice of ingredients used with edible insect diets favouring minimally processed ingredients such as grains or algae-derived supplements, rather than holidic diets favoured for nutritional ecology. Dietary lipids have not received the same attention as protein or carbohydrates in edible insect diets specifically, despite the importance of lipids for endocrine function, reproduction and energy stores (Arien et al., 2020; Guo and Reinhardt, 2020; Leyria et al., 2024). Instead, lipid studies on edible insects centre around the lipid profile of edible insects, either to enhance dietary lipid for other animals, or to decrease the lipid content of edible insects given their potential to have high lipid contents, rather than the effects on the insects themselves (Ajdini et al., 2025; Palupi et al., 2025). Research is transitioning to the importance of macronutrient quality, such as amino acid composition (Piper et al., 2017) and simple versus complex sugars, as well as micronutrients such as vitamins and minerals (Rivera-Pérez et al., 2017). Additionally, physical properties of insect diets can explain some variation in growth and development (Baliota et al., 2024; Muzzatti et al., 2025). Enough data for the best studied species (e.g. yellow mealworm *Tenebrio molitor*) exists to develop diets that can optimise production, however it is complicated to synthesise all these data into a concrete recommendation for ‘the best diet’ (Morales-Ramos et al., 2020). Although optimised diets can theoretically result in optimised production, they can contain high quality (but unsustainable) ingredients like fishmeal, which makes them expensive and unsustainable, and limits scalability (Hawkey et al., 2021). Evaluating diets must consider the physical and nutritional aspects of the feed ingredients, the economic feasibility and the scalability or availability of ingredients if considering alternative ingredients, the environmental impacts of using high production cost ingredients (e.g. fishmeal) and the potential for behavioural self-selection in modulating nutritional intake (Morales-Ramos et al., 2020).

A diet does not necessarily have to be optimised for insects to successfully feed and develop. For example, crickets (*Gryllodes sigillatus*) will feed and successfully grow on diets containing agricultural by-product ingredients (e.g. spent brewers grain) (Kasdorf et al., 2025). There is a wealth of literature demonstrating the potential of black soldier fly larvae (*Hermetica illucens*) to convert organic wastes into high quality protein and frass (Oonincx et al., 2015), but challenges that restrict scalability remain, such as pretreatment of waste streams to increase digestibility (Peguero et al., 2024) and consistently reliable acquisition of high-quality and cost-effective ingredients (Morales-Ramos et al., 2020). Diet is ultimately a highly complex factor in any animal production system, as there are species-specific requirements but also regional restrictions on ingredient quality and availability. Since feed recipes are often considered proprietary, information regarding successful industry diets are not frequently shared. An emerging technique that blends genetics and nutritional ecology could be leveraged by the edible insect industry to rapidly develop high quality strain-specific diets. Exome matching involves designing a diet based on the protein coding regions of an organism's genome. These diets can reduce total food intake while enhancing early life fitness in fruit flies (Piper et al., 2017). Explicit interdisciplinary collaborations between geneticists, nutritionists, and agricultural economists could rapidly adapt this promising approach to insect farming, possibly enhancing yields while reducing costs. Research that quantifies metabolisable energy of diets as well as the energy required for insects to grow and develop in a variety of abiotic conditions (e.g. temperature, humidity) would parametrise quantitative models of insect growth, (e.g. Dynamic Energy Budget models), that could facilitate prediction of optimal conditions for higher production yield of insects (Sousa et al., 2010).

### Behaviour is an indicator and modifier of responses to the abiotic environment and diet

Behaviour is important in a farmed population, even at high rearing densities. In addition to thermoregulation, insect behaviours affect oviposition site selection and reproduction frequency. These choices have downstream consequences for offspring survival and development, as well as for the persistence of populations (Bonebrake et al., 2010). Abiotic factors such as temperature and moisture are modifiers of insect behaviour through microenvironmental effects that interact to determine an individual’s fitness, often in species-specific ways (Notter-Hausmann and Dorn, 2010). As these processes, such as reproduction, can determine the continued success of a farmed population, behaviours can be manipulated for optimal yields. For example, by providing preferred oviposition substrates or thermally varying microhabitats that maximise reproductive success. Understanding these behaviours can aid in designing enclosures that uses a species’ biology for the benefit of mass-rearing.

Artificially high densities created by mass-rearing in small enclosures may disrupt interactions between conspecifics in ways that are not observed in nature. If social interactions or social cues important for intraspecific competition and interactions impact breeding or development, then the breeding or production capacity of a farmed population may be negatively impacted (e.g. through crowding in shelters, drowning of mating calls, competition for food or oviposition sites). High densities can also facilitate increased frequencies of cannibalism (Chang et al., 2023). Potential benefits of cannibalism include additional feeding opportunities facilitating larger sizes or reduced parasite transmission through the consumption of infected individuals (Van Allen et al., 2023). However, these benefits can be highly stage- or sex-dependent and need to outweigh the costs of population loss and economic consequences (Bayoumy and Michaud, 2015). Understanding the drivers and frequency of cannibalism can help with assessing stress responses to the abiotic environment and has important ethical implications for animal welfare but this area of research is still in its infancy (Hansen et al., 2011; Delvendahl et al., 2022). Practical solutions could manage rearing densities by evaluating stocking densities that maximise harvested yields while minimising product loss, and engineering environments that maximise surface area for shelter to avoid crowding and provide multiple feeding and watering points to minimise starvation.

### Social environments drive disease dynamics and pests

Whenever an organism is reared *en masse* in a single location, it presents a rich and abundant resource for other non-target organisms that could be commensal, predatory or pathogenic to the targeted farmed species. However, few pests and pathogens of insects as food and feed facilities have been reported in the literature and the diversity and abundance of non-target species in insect farms are likely underreported. For examples, mealworm farms are susceptible to infestations of pyralid moths (Deruytter et al., 2021), some stored products of mealworms can be infested by beetle pests (Rumbos et al., 2020), and cricket farms may be infested by dermestid beetles (Muzzatti et al., 2025). High-density mass-rearing can provide ideal conditions for the rapid spread of pests and pathogens, yielding devastating consequences for insect farms, especially for cases of diseases caused by species-specific viruses (Bertola and Mutinelli, 2021; Maciel-Vergara et al., 2021). For example, in 2009 a densovirus caused major losses and colony collapses in the USA of the European house cricket, *Acheta domesticus* (Weissman et al., 2012). Such viruses present a real threat to the industry because it is challenging to identify infections, making limiting spread and eradication near impossible. Consequences of these non-target species on yield can also be cryptic. Black soldier fly are susceptible to a wide range of parasites but it is unclear whether they threaten mass-production (Reguzzi et al., 2021; Rojo, 2024). Monitoring and characterising the diversity and consequences of pests and diseases in farmed insect populations will be crucial for protecting the industry against biosecurity threats.

Successful pest management hinges on prevention and transparency. We need to know what pests businesses have dealt with or are currently dealing with, and what approaches are used to prevent, manage and/or control these pests. Partnerships between entomologists and industry leaders would be crucial in developing best practices for pest management. Basic prevention begins with maintaining sanitation and hygiene which benefits the broader workplace as some of these pests present allergen risks to farm employees (Deruytter et al., 2021). However, once an outbreak of disease or pests is detected, best practices for how to respond and manage are less certain. Quarantine can be effective but is highly disruptive to operations. Pesticides are not ideal when they also adversely affect the health and wellness of the farmed insect. Transparency in how established industry leaders manage pests will assist emerging leaders with their own pest management practices.

Active monitoring for emerging threats can include metagenomics sequencing of infected individuals and eDNA approaches (Allen et al., 2021; Lim et al., 2024). However, the adoption of such technology can lag behind the development of a farming operation. There is a need to consider biosecurity during the designing and planning phases for a facility. All these approaches will incur added costs to the operations of a farm, but not doing so may risk *ad hoc* pest management strategies that are ineffective. Increasing our knowledge of the diversity and effects of pests present on insect farms will fill our knowledge gaps and allow for more informed planning and design of insect farms for pest and disease management. Pest management inside insect farms is a field of study ripe for integrative and cross-discipline research that strike a delicate balance between eradicating unwanted species without detrimental impacts on the farmed species.

### Genes underpin biological phenotypes advantageous to farming

Insects make up an estimated 55% of extant global biodiversity (Bánki et al., 2025), yet only 815 insect genomes are freely available (Mei et al., 2022). After recent interest in edible insects, four of the major farmed species in the northern hemisphere have chromosome level assemblies available on NCBI: *Hermetica illucens* (black soldier fly), *Tenebrio molitor* (yellow mealworm), *Gryllodes sigillatus* (decorated cricket), and *Acheta domesticus* (house cricket) (https://www.ncbi.nlm.nih.gov/datasets/genome/; accessed 29 May 2025). Other species of economic interest for insect farming available include *Gryllus assimilis* (field cricket) and *Bombyx mori* (silkworm). Yet, many more edible insect species have not been sequenced. In contrast, all vertebrate livestock species have chromosome level assemblies (Hubert et al., 2024), and over 1800 plant genomes are available (https://www.plabipd.de/pubplant_main.html, accessed 22 Jan 2025), with many horticulturally important species represented (Chen et al., 2019). A lack of investment in insect genetics in general means that many species still have not been identified (Li and Wiens, 2023) and we know very little about population structure and adaptation for the species we do know (Beaurepaire et al., 2024). Understanding population structure of farmed insects harvested from the wild for the food and feed industry is necessary to ensure effective and sustainable large-scale production. For example, rearing populations can be established from wild progenitors, but without management of inbreeding consequences, rapid collapse of the population can occur within five generations (Rhode et al., 2020). Similarly, if operations establish their stock from other established farms/suppliers, then the genetic provenance of the source population may be unknown. Genetic tools can be developed to manage inbreeding and ensure appropriate stock is used for supplementing. Genetic tools are also necessary to identify appropriate stock, as introducing individuals that are too distantly related can disrupt local adaptation leading to negative fitness consequences because of outbreeding depression.

In general, the use of genomics in the food and feed industry is still in its infancy for the majority of species and increased investment will be required to ensure predictable and sustainable production (Oudijk et al., 2025). More specifically, tools like genomic selection (Goddard and Hayes, 2007) can contribute to identifying and selecting for favourable traits which can lead to increased quality of the final product, improving health and stability of the population, and decreasing the amount of time to harvest (Eriksson and Picard, 2021). However, the use of genomic selection has not yet been applied, though characteristics to target selection have been identified (e.g. Nakajima and Ogura, 2022), and a recent study has estimated heritabilities for important phenotypic traits in the house fly (*Musca domestica*) (Hansen et al., 2024). Explicit investment into genomic selection programs tailored specifically for commonly farmed insects (e.g. black soldier fly, mealworms, crickets) is essential. Applying genomic selection frameworks already proven effective in other livestock could rapidly improve traits such as growth rate, feed efficiency, disease resistance, and stress tolerance (Eriksson and Picard, 2021; Hansen et al., 2024).

### Weighing yield improvements against product demand and consumer responses

While many of the interdisciplinary questions discussed herein involve significant financial investments to scale up production, these costs are ultimately dependent on the profitability of businesses or investors to invest or reinvest in improving farming operations to achieve economic sustainability (long-term viability) (Biteau et al., 2024). Product demand drives much of these economics. There is an established but relatively small market for crickets, black soldier fly larvae and mealworms as a high-protein food for captive reptiles, birds, fish, and some small mammals. Use of insect-derived ingredients in food for more popular companion animals, namely dogs and cats, is growing and represents a major potential market opportunity for the insect sector, particularly for premium pet foods that contain more animal- and insect-based protein (Valdés et al., 2022). Pet foods sold in Canada, the UK, Australia, and many European countries already contain insect ingredients, and the US Food and Drug Authority recently approved the use of mealworm protein in dog foods (Ÿnsect, 2024). While the relative environmental sustainability of insect-based ingredients is a selling point (van Huis and Oonincx, 2017), consumer acceptance of insects as pet food can be a barrier, especially in western cultures. Higa et al., 2021 found that American dog owners were more likely to be willing to feed their dogs treats made with insect flour (stated willingness of approximately 50 on a 100-point scale) relative to whole dried insects (approximately 30 points).

These findings mirror patterns of human acceptability for direct consumption of insects as food, which tends to be quite low in North America. While 26–27% of participants in the study by Higa et al., 2021 said they would be comfortable consuming products made with a modest percentage of insect ingredients, only 12% of subjects indicated they would be willing to try whole roasted insects in the absence of a financial incentive. Other studies have found similar levels of consumer acceptance in contexts as diverse as India (Ruby et al., 2015), the Netherlands (Tan et al., 2016), and Greece (Giotis and Drichoutis, 2021). Higher acceptance of foods with insect ingredients was observed in a sample of people in rural Nigeria who were already insect consumers (Awobusuyi et al., 2020), but such populations are relatively small. One study compared consumers’ ratings of unlabeled burgers that included ground mealworms, against pure beef and pure lentil burgers, and found mealworm burgers were rated as intermediate between beef and lentil burgers overall, though men’s acceptance of mealworm burgers was significantly higher than women’s (Caparros Megido et al., 2016).

A larger potential market opportunity than the pet food market would be the use of insect meal as a full or partial replacement of other protein sources in livestock feed, namely soybean meal (primarily used in poultry production) and fish meal (primarily used in aquaculture). However, recent analyses indicate that insect-derived products are unlikely to be price-competitive with either of these alternatives in the foreseeable future. Long-term (post 2030) projections of the price per metric ton of insect protein at scale range from 1600 to 2700 USD, whereas fishmeal prices are expected to remain relatively constant at around 1250 USD by 2031 (FAO, 2022; Biteau et al., 2024). Further, consumers do not appear willing to pay a significant price premium for insect-fed livestock (Biteau et al., 2024).

Rearing insects involves major production costs and a long production chain that make them economically uncompetitive against common protein sources such as fishmeal and soybean meal (Biteau et al., 2024). Leipertz et al., 2024 modelled the costs of defatted larvae meal in the Netherlands and estimated the cost of production at €5116/tDM, compared to a cost of delivering equivalent nutrition from fishmeal of €1296 or €499 from soybean meal. In these models, raw substrate (feed) constituted the most important production cost, at 42% of the total, with capital costs (building and inventory) second at 32% (Leipertz et al., 2024). This cost structure implies that improvements in production efficiency will not be sufficient to achieve economic viability. Reaching cost equivalence with fishmeal would require either negative input prices or significant revenue from by-products (e.g. frass). However, potential low-cost substrates such as manure would introduce food safety risks and require drying steps or mixing with dry inputs, increasing their costs. On the by-product side, the price of frass would need to reach €1175/tDM for larvae meal to reach cost-equivalency with fishmeal (Leipertz et al., 2024). An existing frass product sells for €854/tDM, and Leipertz et al., 2024 modelled frass prices at scale as €89/tDM. Also noteworthy are the contributions of labour (9%) and heating (6%) to total production costs that are not equivalent to soybean farming or fishing (Biteau et al., 2024; Leipertz et al., 2024). Lower costs for these inputs in tropical climates may imply that northern countries have a comparative disadvantage in the production of insects and may implicitly increase the environmental impacts of insect production (Oonincx and de Boer, 2012; Biteau et al., 2024).

Published information on the relative costs of animal-based ingredients versus insect ingredients in pet foods is not available but is likely to be a major factor in the extent to which insect-based ingredients scale up beyond premium pet food products marketed as sustainable. Analysis of offal prices reported by the US Department of Agriculture indicates that the average price per tonne of offal from steers is in the range of $3500 to $7000 US, depending on whether blood meal and bone meal are included (authors’ calculations using by-product drop value for steers (USDA, 2025)). This suggests that insect ingredients could potentially be competitive animal protein sources in pet food. Further analysis based on the specific animal ingredients in pet foods and the nutritional properties of insect versus animal ingredients is needed to answer this question with greater certainty.

### Potentials of interdisciplinary actions: Opportunities and challenges

Bridging siloed disciplines brings the potential for responding to opportunities and challenges of the edible insect industry in interdisciplinary ways (Fig. 1A). We highlight this opportunity in two hypothetical examples. First, there is a unique opportunity at the intersection of biology, engineering, and technology to develop systems for precision insect farming that can address the problem of scale. High-technology approaches have benefitted agriculture in closed facilities, such as greenhouses, by providing automatic, controlled environment systems (Lee et al., 2024), and AI-driven detection of insect crop pests (Popescu et al., 2023; Wang et al., 2023). The same principles can be translated towards closed insect rearing facilities. In these systems, co-operation between engineers and biologists can integrate biological insights with technological solutions to refine the design and construction of a facility that can respond to the insect’s needs or behaviours in real time or increase automation (Fig. 1B). Examples include environmental control systems to mitigate the risk of unintended environmental fluctuations or modify abiotic conditions to the benefit of specific biological processes such as reproduction, sensors to monitor condition of food, water and density in enclosures, and layouts or materials that maximise biosecurity. The data collected by sensors can be analysed to identify intersecting areas that are synchronously affected by a common factor, for example increasing temperature can influence insect development to improve yield but can also have consequences for the spread of disease (Fig. 1B). This relationship is inherently interdisciplinary; engineers require biologists to communicate needs and preferences of the organisms, whereas biologists require engineers for unique and creative solutions that fit within the logistics of a mass-production facility. High-technology approaches are not suitable for all contexts of insect farming but a controlled environment has added benefits for ensuring the comfort, health, and safety of workers especially if rearing insects at temperatures above human comfort levels. Ultimately, given farming operations seek to make profit, any suggested recommendations to increase scale or yield need to be weighed against upfront or long-term costs. Evaluating changes to production involves communicating biological information to economists to construct bioeconomic models that can aid practitioners in making informed decisions (González and Neff, 2026) (Fig. 1B).

Second, an unexpected pest outbreak demonstrates an interdisciplinary feedback loop in a volatile situation. The establishment of a non-target species involves biosecurity (engineering), favourable biotic conditions for a pest (biology) and ineffective monitoring (engineering) (Fig. 1C). The consequences of these pests on target species life history (biology) would affect yield and lead to improved monitoring and biosecurity, as well as changes to operational materials (Fig. 1C). In this example, a positive outcome is to improve preventative measures through communication between biologists and engineers to prevent future outbreaks and manage risk. Without a rapid response, the outcomes can be devastating, including a crash of the insect population, a complete sterilisation of the facility and a loss of production. Successful interdisciplinary partnerships depend on fast, open communication that can facilitate rapid responses to emerging challenges and volatile situations from local to global scales.

## Conclusions

Co-operative research is central to the edible insect industry. Here we suggested ways that different disciplines can intersect to ask sophisticated questions, answers for which are needed to advance the edible insect industry. Many of our outstanding research questions lie at the intersection of two or more disciplines, including others that were not covered here such as the societal aspects of insect farming (Mishyna et al., 2020). Interdisciplinary research within academia is not without challenges. Identifying the overarching goals based on common research interests is important to overcome this challenge. Research centres and consortiums are an example of successful interdisciplinary science, such as ICIPE, Kenya (https://www.icipe.org/). The next step to guide future research directions is to establish best practices policies, as has been suggested for the cricket, *Acheta domesticus* (VanPeer et al., 2024). There is an opportunity to draw expertise and lessons from other fields of applied science. Examples of translational science in other applied fields include integrating animal physiology into pollination or sterile insect technique practices (Sinclair et al., 2022). Co-operation between stakeholders of the edible insect industry is the core of research. The partnership between industry and academia has driven an exponential increase in research outputs as the industry has developed (Baiano, 2020). These partnerships can streamline goal setting and direct research to meet the needs of the industry. However, balancing the goals of academia and the needs of the industry remains a major hurdle for collaboration. Publishing with an industrial partner can become a conflict of interest that should be transparent in the publication record.

More recently, formal co-operations between industry, governments and academia at a regional or international level have emerged. These initiatives ensure the consistent advancement of the industry and are important for establishing and strengthening relationships in conjunction with professional conferences, such as Insects to Feed the World (van Huis et al., 2015). Examples include multi-organisational consortiums such as MINIstock (Robinson et al., 2024), the International Platform of Insects for Food and Feed (IPIFF, https://ipiff.org), the Center for Environmental Sustainability through Insect Farming (www.insectcenter.org), the North American Coalition for Insect Agriculture (https://nacia.org), or professional societies such as the Academic Society for Insects As Food and Feed (https://asiff.org). Governments play an important role in establishing policies and regulatory frameworks for the industry and for the benefit of consumers from local to international scales (Tomberlin et al., 2022; Żuk-Gołaszewska et al., 2022). For example, the Insect Regulatory Framework developed by the Singapore Food Agency sets import, export and operational guidelines for local businesses (Stevens and Ruperti, 2024). In summary, interdisciplinary collaboration is essential in the edible insect industry. These relationships span levels of organisation from academia to government from a local to international level. Each of these relationships informs research into edible insects in multiple ways. Together, the edible insect industry can grow to meet the global demands for insect protein.

## CRediT authorship contribution statement

**Jacinta D. Kong:** Project administration, Conceptualization, Writing – original draft, Writing – review & editing. **Matthew J. Muzzatti:** Conceptualization, Writing – original draft, Writing – review & editing. **Catherine I. Cullingham:** Funding acquisition, Conceptualization, Writing – original draft, Writing – review & editing. **Vivian Hoffmann:** Funding acquisition, Conceptualization, Writing – original draft, Writing – review & editing. **Susan M. Bertram:** Funding acquisition, Conceptualization, Writing – original draft, Writing – review & editing. **Heath A. MacMillan:** Visualization, Funding acquisition, Conceptualization, Writing – original draft, Writing – review & editing.

## Declaration of competing interest

The authors declare the following financial interests/personal relationships which may be considered as potential competing interests:

Jacinta Kong reports financial support was provided by NSERC Alliance and Mitacs Accelerate. Heath MacMillan reports financial support was provided by NSERC Alliance and Mitacs Accelerate. Susan Bertram reports financial support was provided by NSERC Alliance and Mitacs Accelerate. Heath MacMillan reports financial support was provided by NSERC Discovery Grant. Susan Bertram reports financial support was provided by NSERC Discovery Grant. Jacinta Kong reports a relationship with Aspire Food Group that includes: funding grants. Heath MacMillan reports a relationship with Aspire Food Group that includes: funding grants. Susan Bertram reports a relationship with Aspire Food Group that includes: funding grants. Jacinta Kong reports a relationship with Entomo Farms that includes: funding grants. Susan Bertram reports a relationship with Entomo Farms that includes: funding grants. Heath MacMillan reports a relationship with Entomo Farms that includes: funding grants. Matthew Muzzatti reports a relationship with Aspire Food Group that includes: funding grants. Matthew Muzzatti reports a relationship with Entomo Farms that includes: funding grants. Matthew Muzzatti reports a relationship with Bug Mars that includes: funding grants. Jacinta Kong reports a relationship with Bug Mars that includes: funding grants. Heath MacMillan reports a relationship with Bug Mars that includes: funding grants. Susan Betram reports a relationship with Bug Mars that includes: funding grants. If there are other authors, they declare that they have no known competing financial interests or personal relationships that could have appeared to influence the work reported in this paper.
